# Infantile Hemangioma Localized in the Levator Aponeurosis-Müller’s Muscle Complex

**DOI:** 10.7759/cureus.73716

**Published:** 2024-11-15

**Authors:** Lorenzo Ripetta, Jonnah Kristina C Teope, Hidetaka Miyazaki, Yasuhiro Takahashi

**Affiliations:** 1 Oculoplastic, Orbital, and Lacrimal Surgery, Aichi Medical University Hospital, Nagakute, JPN

**Keywords:** excision, infantile hemangioma, levator aponeurosis, localization, müller's muscle

## Abstract

A two-month-old male infant presented with a soft palpable mass on his left upper eyelid. Initial management consisted of watchful observation followed by administration of β-blocker eyedrops on the eight-month check-up when a purple subconjunctival mass was observed during eyelid eversion, suggestive of an infantile hemangioma. At the three-year follow-up, since it was observed that the treatment did not reduce the size of the mass, an excisional biopsy was performed at the request of the mother. During surgery, the mass was identified in the levator aponeurosis-Müller’s muscle complex, between the tarsus and the junction of the levator aponeurosis and orbital septum. Pathological examination and immunohistochemical staining confirmed the diagnosis of infantile hemangioma. Symmetric eyelid position was achieved with no recurrence at the three-month follow-up.

## Introduction

Infantile hemangiomas are the most common tumors of childhood, being found in approximately 4-5% of infants [1]. They are characterized as superficial, deep, or mixed, and as focal, multifocal, or segmental [2]. Superficial lesions often present as a strawberry nevus, while deeper ones show as a dark reddish or bluish discoloration [3]. Focal infantile hemangiomas are localized lesions that arise from a single point, whereas multifocal and segmental lesions occur at multiple sites and involve larger areas, respectively [2].

Infantile periocular hemangiomas are focal lesions reported in 1 in 1,586 live births, with the highest prevalence localized on the unilateral upper eyelid [4]. These lesions are known to originate in the preseptal area and then invade the extraconal and intraconal space, and not the other way around [5].

Herein, we report an unusual case of deep focal infantile hemangioma originating in the levator aponeurosis-Müller’s muscle complex, between the tarsus and the junction of the levator aponeurosis and orbital septum.

## Case presentation

A two-month-old male infant presented with a mass in the left upper eyelid. His mother noticed the mass two weeks after his birth. No other lesions were noted. His family history was unremarkable.

On initial examination, a soft, immobile mass was palpable in the left upper eyelid. There was no erythema of the eyelid. Margin reflex distance-1 (MRD-1) could not be measured because the patient did not open his eyes.

The patient was initially managed with watchful observation. At an eight-month follow-up, a purplish subconjunctival mass was observed upon upper eyelid eversion. An infantile hemangioma was then suspected. β-blocker eye drop was administered, but the mass did not reduce in size.

**Figure 1:**
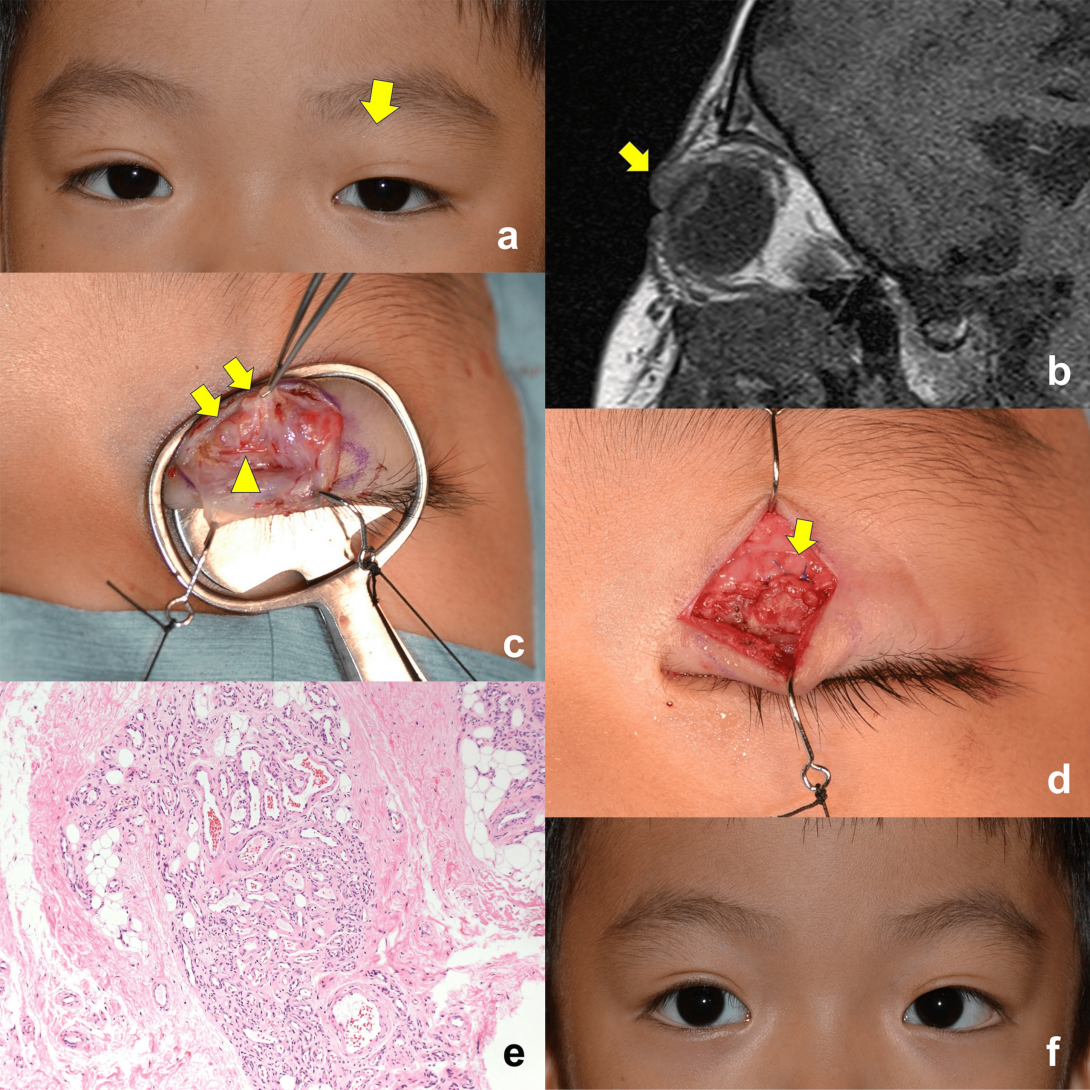
Photos of the case. a. A face photo taken on the day of surgery. Margin reflex distance-1 was 3.0 mm on both sides. The arrow indicates the lesion. b. A sagittal T1-weighted magnetic resonance image showing a low-to-isointense upper eyelid mass (arrow). c, d. Photos taken intraoperatively. c. The lesion was found in the levator aponeurosis (arrows). The peripheral artery (arrowhead) was observed under the lesion. d. The aponeurosis (arrow) was re-attached. e. Pathological examination showing lobulated proliferation of small vessels (hematoxylin and eosin staining; magnification, x100). f. A face photo taken three months after surgery showing a symmetric upper eyelid position.

At three-year follow-up, visual acuity was 0.1 in both eyes, and the patient did not have refractive errors. MRD-1 was 3.0 mm on both sides (Figure 1a). Surgical excision was scheduled at his mother’s request to rule out malignancy. Magnetic resonance imaging taken one day before surgery showed a mass, sized 3 x 7 x 7 mm, under the orbicularis oculi muscle on the left side (Figure 1b). The mass was low-to-isointense on T1-weighted images and iso-to-high-intense on T2-weighted images. Surgical excision was performed under general anesthesia. After an eyelid crease incision, the mass was identified in the levator aponeurosis- Müller’s muscle complex, between the tarsus and the junction of the levator aponeurosis and orbital septum (white line) (Figure 1c). During the dissection of the mass, the peripheral artery was found under the mass (Figure 1c). After complete excision of the mass, the levator aponeurosis was re-attached to the tarsus (Figure 1d).

Pathological examination showed lobulated proliferation of small vessels lined by endothelial cells (Figure 1e). Immunohistochemical stains for Glut1, CD31, and CD34 were positive. Pathological findings corresponded to infantile hemangioma.

At three-month follow-up, the mass did not recur. Visual acuity was 1.0 in the right eye and 0.9 in the left eye. The upper eyelid position was symmetric (Figure 1f). His eye position was orthophoria.

## Discussion

We report an unusual presentation of periocular infantile hemangioma due to the site of origin of the tumor. Periocular hemangiomas typically originate in the preseptal area and extend into the orbital space through finger-like posterior projections [5]. This patient had an isolated infantile hemangioma located in the levator aponeurosis-Müller’s muscle complex, between the tarsus and white line (newly classified as the pre-fascial part, not the preseptal part) [6]. The purplish subconjunctival mass observed upon eversion of the upper eyelid displayed the characteristic appearance of a deep localized infantile hemangioma. However, contrary to the usual pattern, magnetic resonance imaging confirmed that this hemangioma was located beneath the orbicularis oculi muscle, with no posterior extension. This finding was further validated during surgery, where the mass was identified in the levator aponeurosis-Müller’s muscle complex, with the peripheral artery under the lesion. There was no preseptal component noted.

The present case did not show ptosis or refractive error despite the location of the lesion. A small, soft tumor located just above the upper tarsus may not have interrupted the eyelid opening and compressed the corneal surface. However, prolonged invasion of the levator aponeurosis-Muller’s muscle complex may result in a fatty, atrophic muscle similar to true congenital ptosis. In addition, approximately 50-70% of infantile hemangiomas that spontaneously regress leave behind residual skin changes, including telangiectasia, redundant skin, dyspigmentation, or scarring [7]. Both superficial and deep periocular infantile capillary hemangiomas have been shown to respond to the use of topical β-blockers such as timolol-maleate drops at 0.5% while having fewer side effects such as bradycardia, hypotension, and bronchial hyperactivity when compared to systemic β-blockers, which is why they were employed as the first-line treatment for this case [2,8]. While treatments such as oral β-blockers, intralesional steroids, and sclerosing agents are also viable, granting the mother’s request for surgical excision seemed to be a safe option after the patient showed no improvement with the topical β-blockers in the three-year follow-up [2].

## Conclusions

We presented a case of infantile periocular hemangioma originating from an unusual location. Although infantile hemangiomas are generally diagnosed based on history and clinical examination, imaging is recommended to accurately assess the extent and location of the lesion, as it may have a post-septal origin. This understanding is important for guiding the appropriate treatment approach.
